# SOCIAL ISOLATION MEASUREMENT AND OLDER ADULT HEALTH: A REVIEW

**DOI:** 10.1093/geroni/igz038.3050

**Published:** 2019-11-08

**Authors:** Janet S Pohl

**Affiliations:** Betty Irene Moore School of Nursing at University of California Davis, Sacramento, California, United States

## Abstract

The objectives were to examine social isolation research literature, investigate reports of associations with health, and explore the numerous approaches to operationalize social isolation in gerontological research. While associated with negative health outcomes and mortality, the interpretation of social isolation research is hampered by a lack of conceptual clarity and the use of numerous ad hoc measures of the concept. A systematic search was conducted for published empiric studies regarding social isolation health outcomes in older adult samples. The electronic databases: Medline, CINAHL, and PsycINFO were utilized. Reports including social isolation as an independent variable and health outcomes at the individual level were extracted. Of 2,614 studies initially identified, 14 met study criteria. Study outcomes recognized smoking cessation, sleep disruption, inadequate diet, risk for malnutrition, health-related quality of life, subjective well-being, cognitive function, psychological distress, depression, functional decline, stroke, myocardial infarction, and mortality as related to social isolation. Measurement strategies revealed numerous definitions of social isolation reporting to evaluate objective and subjective social isolation, loneliness, engagement, social disconnectedness, and perceived isolation. Measures utilized: eight ad hoc, three versions of the Lubben Social Network Scale, two versions of the Social Network Index, and one question from the Rand Social Battery. Continuing to develop knowledge regarding the predictive power of social isolation on health is important for the care of older adults. Distinguishing social isolation from related but distinctly different social concepts will facilitate the forward movement of the science. Reliably measuring social isolation will enable the comparison of results across studies.

